# Cloning, Characterization and Functional Analysis of the *LtuPTOX* Gene, a Homologue of *Arabidopsis thaliana* *IMMUTANS* Derived from *Liriodendron tulipifera*

**DOI:** 10.3390/genes10110878

**Published:** 2019-11-01

**Authors:** Ziyuan Hao, Yaxian Zong, Huanhuan Liu, Zhonghua Tu, Huogen Li

**Affiliations:** 1 Key Laboratory of Forest Genetics & Biotechnology of Ministry of Education, Co-Innovation Center for Sustainable Forestry in Southern China, Nanjing Forestry University, Nanjing 210037, China; lxhzy1992@163.com (Z.H.); zyx269833@163.com (Y.Z.); lhh91@njfu.edu.cn (H.L.); zhonghuatu@njfu.edu.cn (Z.T.); 2 College of Forestry, Nanjing Forestry University, Nanjing 210037, China

**Keywords:** *L. tulipifera*, tepal, carotenoid biosynthesis, *LtuPTOX*, functional analysis

## Abstract

Flower colour and colour patterns are crucial traits for ornamental species; thus, a comprehensive understanding of their genetic basis is extremely significant for plant breeders. The tulip tree (*Liriodendron tulipifera* Linn.) is well known for its flowers, odd leave shape and tree form. However, the genetic basis of its colour inheritance remains unknown. In this study, a putative plastid terminal oxidase gene *(LtuPTOX)* was identified from *L. tulipifera* based on multiple databases of differentially expressed genes at various developmental stages. Then, the full-length cDNA of *LtuPTOX* was derived from tepals and leaves using RACE (rapid amplification of cDNA ends) approaches. Furthermore, gene structure and phylogenetic analyses of PTOX as well as AOXs (alternative oxidases), another highly similar homologue in the AOX family, were used to distinguish between the two subfamilies of genes. In addition, transient transformation and qPCR methods were used to determine the subcellular localization and tissue expression pattern of the *LtuPTOX* gene. Moreover, the expression of *LtuPTOX* as well as pigment contents was investigated to illustrate the function of this gene during the formation of orange bands on petals. The results showed that the *LtuPTOX* gene encodes a 358-aa protein that contains a complete AOX domain (PF01786). Accordingly, the *Liriodendron* *PTOX* and *AOX* genes were identified as only paralogs since they were rather similar in sequence. LtuPTOX showed chloroplast localization and was expressed in coloured organs such as petals and leaves. Additionally, an increasing pattern of *LtuPTOX* transcripts leads to carotenoid accumulation on the orange-band during flower bud development. Taken together, our results suggest that *LtuPTOX* is involved in petal carotenoid metabolism and orange band formation in *L. tulipifera*. The identification of this potentially involved gene will lay a foundation for further uncovering the genetic basis of flower colour in *L. tulipifera*.

## 1. Introduction

Flower colour is an important trait for ornamental species, as brightly coloured flowers are attractive to people. Additionally, colour patterns and colour spots play an essential role in attracting pollinators to insect-pollinated flowers, helping to improve the fitness of seeds and gene flow within species [1,2,3]. The response of pollinators to flower color is closely related to plant development and vigour [4]. Pigments in the petals have been extensively studied in many species [5,6], whereas the most important pigments in flowers are flavonoids, carotenoids and alkaloids [7].

**Figure 1:**
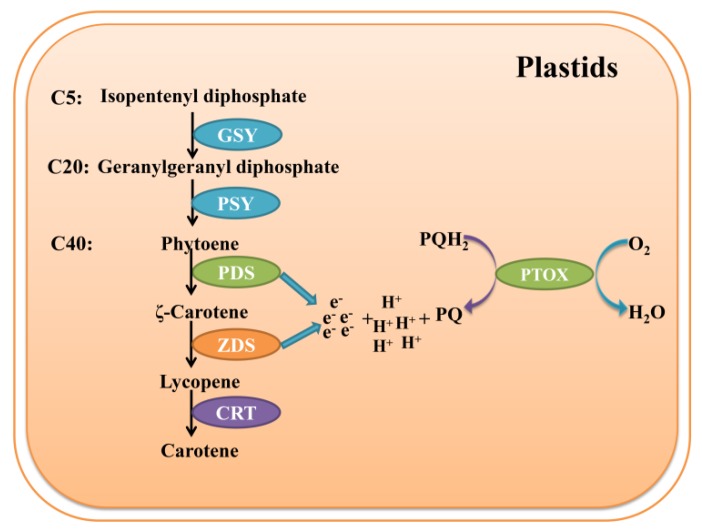
PTOX participates in the process of carotenoid metabolism in plant plastids [9,14,21]. PTOX, plastid terminal oxidase; PQ, plastoquinone; PQH_2_, reduced plastoquinone; GSY, geranylgeranyl pyrophosphate synthase; PSY, phytoene synthase; PDS, phytoene desaturase; ZDS, zeta-carotene desaturase; CRT, carotenoid isomerase.

In recent decades, many studies have focused on the formation of petal colour in plants. In particular, the synthesis and degradation of anthocyanins have been illustrated [7], yet the genetic mechanism of carotenoid metabolism remains unclear. One of the reasons for this ambiguity is that carotenoids are a subgroup of isoprenoid compounds that contain over 1158 isoforms [8]. Moreover, the genetic regulatory and metabolic pathways of carotenoids are not unique to C40 and other carotenoid categories. Nevertheless, the precursor for the synthesis of almost all carotenoids is isopentenyl diphosphate, an organic substance containing five carbon atoms [9,10]. Multistage precursors involve a set of catalytic enzymes (including geranylgeranyl pyrophosphate (GGPP) synthase, phytoene synthase, phytoene desaturase (PDS), zeta-carotene desaturase (ZDS) and carotenoid isomerase (CRTISO), among others; see Figure 1), that are ultimately converted to carotene [9]. In addition, some co-factors such as phytochromes [11,12], electron mediators [13,14] and transcription factors [15] also participate in the synthesis of carotenoids.

Plastid terminal oxidase (PTOX) is a terminal oxidase involved in chlororespiration. The discovery of this gene took more than half a century. Diner and Mauzerall first revealed the positive feedback of oxygen in the photooxidation system almost fifty years ago [16]. Interestingly, this discovery indicated that there is a novel oxygen-dependent electron transport chain (ETC) in chloroplasts. Subsequently, in the 1980s, Bennoun proved that a photorespiration chain, which was similar to the respiratory ETC in mitochondria, also existed in the chloroplasts in plants [17]. In 1999, the *immutans* mutant of *Arabidopsis thaliana* brought attention to the PTOX gene [18,19]. Green and white stripes on the leaves of *immutans* confused researchers because they were not caused by the loss of enzymes in the chlorophyll synthesis pathway; the white sectors contained few carotenoids but accumulated phytoene [20]. The same pattern was also observed in the *ghost* mutant of tomato, with faded fruits, and further studies revealed that this abnormal phenotype was caused by the loss of PTOX gene function. In contrast to other enzymes in the carotenoid metabolic pathway, PTOX did not directly accelerate the conversion of intermediate products but accepted the electrons from PDS and ZDS and then delivered them as soon as possible (Figure 1) [21]. Importantly, the absence of PTOX combined with the *IMMUTANS* and *GHOST* mutations almost impeded carotenoid accumulation. Hence, PTOX is considered an indispensable cofactor in the synthesis of carotenoids.

As a cyanide-sensitive oxidase, PTOX was frequently used to compare with another ubiquinol oxidase AOX (alternative oxidase). PTOX and AOX sequences are fairly similar and also regarded as homologues in many other studies [22]. One of the reasons is that they are both SHAM (Salicylhydroxamic acid) sensitive. Besides, the two subfamilies share the same domain of AOX (PF01786) in the C-terminus including two “ExxH” iron-binding motifs and a E(x)_6_Y motif as described by Carol and Kuntz [9]. Although they are similar in sequence and domains, nevertheless, gene structure analysis would give us a viewpoint to distinguish these two proteins. Additionally, PTOXs mainly exist in plastids, especially in chloroplast, while AOXs are merely found in the inner membrane of mitochondria in accordance with current reports.

*L. tulipifera* is well known as an ornamental tree species due to its brightly colored flowers, odd leaf shape and graceful tree form. The tulip-like color and shape of the flowers give *L. tulipifera* another name: “tulip tree”. As a result, *Liriodendron* is often used for courtyard greening and street embellishment. In recent years, the disadvantages of other ornamental trees are increasingly exposed, i.e., clouds of poplar and planetree catkins; hence, the tulip tree has become incredibly popular in China. *L. tulipifera* originated in North America and was introduced to China decades ago to improve the adaptability and wood yield of *Liriodendron chinense*, a related species native to southern China. Studies of *L. tulipifera* have focused on its origin and evolution [23,24], the development of its flowers and nectaries [25] and its interspecific breeding, among other topics in the past years. Accordingly, attracted to the color and color patterns on the petals, specifically the orange band lying in the nectary area, Demuth also demonstrated that the main pigments of this area of *L. tulipifera* were carotenoids [26]. Even though the orange band is essential for aesthetic purposes and facilitating pollination, the genetic mechanism underlying the formation of this band remains unclear.

Inspired by previous research, we screened the candidate carotenoid synthesis gene *LtuPTOX* based on multiple databases. Moreover, the full-size cDNA of *LtuPTOX* was derived from *L. tulipifera* by rapid amplification of cDNA ends (RACE), and a subcellular localization assay and development-specific expression pattern analysis were carried out. Overall, this study revealed that the *LtuPTOX* gene participates in the synthesis of carotenoids and the development of the orange band on petals, which will be beneficial for characterizing the mechanism of flower color formation in *L. tulipifera*.

## 2. Materials and Methods

### 2.1. Plant Materials

In early summer 2016, several tissues and organs, including leaves, flower buds, petals, stamens, pistils, leaf buds, flower buds and young stems, were removed from a living adult plant of *L. tulipifera* (seeds originating from South Carolina, USA), immediately frozen in liquid nitrogen, brought back to the laboratory and stored at −80 °C. Petals and leaves were used for gene isolation, and all the other tissues and organs were used to investigate the tissue-specific expression patterns of *LtuPTOX*. Petals at different developmental stages were obtained during the flowering period (from April to May 2017). All the *Liriodendron* trees were planted at Xiashu Forest Farm, which is attached to Nanjing Forestry University, Jiangsu Province, China. The *Nicotiana benthamiana* material used for the subcellular localization assay was obtained from the Key Laboratory of Forest Genetics and Biotechnology, Ministry of Education, Nanjing Forestry University. Trans T1 and GV3101 strains were used for gene cloning and transient expression, respectively.

Tobacco seeds were sterilized with 10% NaClO for 10 min and then sown in a nutrition matrix in an artificial illumination incubator under appropriate conditions (22 °C, with a 14-h light and 10-h dark photoperiod). Approximately 30 days after sowing, tobacco seedlings in the eight-leaf stage were used for transient transformation and subcellular localization analysis.

### 2.2. Identification and Isolation of the Full-Length LcPTOX Gene in L. tulipifera

Limited by the incomplete annotations of the *Liriodendron* genome database, we filtered *LtuPTOX*-homologous genes from multiple databases, including the classical expressed sequence tag (EST) database [27] of *L. tulipifera* (AAGP, http://jlmwiki.plantbio.uga.edu/aagp/), and a local RNA-seq database of distinct flowering stages obtained by Illumina Novaseq6000 (presented in another article under review). Because the EST fragments were short and incomplete, we mapped all the *LtuPTOX* fragments obtained from the EST database to the local RNA-seq differentially expressed gene (DEG) database and the recently released genome database of *L. chinense* [24]. Redundant sequences were removed, differential expression at distinct stages was considered, and a putative *LtuPTOX* gene (partial sequence, not complete cDNA) was obtained for further study. To obtain the full sequence of *LtuPTOX*, we further developed a RACE assay to amplify the unknown sequence. Primers used for isolating the 5′ and 3′ untranslated regions of *LtuPTOX* have been listed in the Table A1 (Appendix A), and sequences of the 5′ and 3′ untranslated regions were assembled by DNAstar software.

### 2.3. Structural and Functional Identification of LtuPTOX by Bioinformatic Approaches

To preliminary understand the function and properties of LtuPTOX, a series of software and approaches were used for analysis. The LtuPTOX protein sequence was obtained by submitting the full-length cDNA of the LtuPTOX gene to an online tool (https://www.ncbi.nlm.nih.gov/orffinder/) for open reading frame (ORF) prediction. The physicochemical properties of the LtuPTOX protein were analyzed using ExPASy ProtParam online software (https://web.expasy.org/protparam/). Moreover, the conserved domain of the LtuPTOX protein was determined by Pfam (http://pfam.xfam.org/), and tertiary structure prediction was performed with I-TISSER software [28]. Location and structural information of the LtuPTOX gene were further obtained by genome-wide identification. Accordingly, the differences in gene structure between AOX and PTOX were assessed using GSDS 2.0 software [29], since the genes have been considered homologous genes over the past years. Additionally, sequences were aligned with those from other species and visualized by ESPrint 3.0 software [30]. We further performed a standard phylogenetic analysis, using MAFFT software [31,32] for multiple sequence alignment and Gblocks 0.91 software [33] for conserved domain selection. Finally, an optimal amino acid substitution model was selected using MEGA7 software, and with this optimum model, the phylogenetic relationships were demonstrated with a neighbor-joining (NJ) tree.

### 2.4. Transient Expression and Subcellular Localization of the LtuPTOX Gene

The pCAMBIA1302 vector was digested by NcoI and SpeI quick-cut enzymes (1620 and 1631, TAKARA, Dalian, China), and then we inserted the complete ORF sequence into the digested pCAMBIA1302 vector. Furthermore, the raw pCAMBIA1302 vector and the recombinant vector carrying the *35S:LcPTOX-GFP* gene were transferred into the GV3101 *Agrobacterium* strain. With the P19 helper vector, two expression stains were injected into the lower epidermis of tobacco leaves. After 2–3 days of incubation at 23 °C, the lower epidermis of infected tobacco leaves was removed carefully and placed on a glass slide, and then we observed the slices at distinct wavelengths using a laser confocal microscope (LSM710, Carl Zeiss, Germany).

### 2.5. Expression Pattern of the LtuPTOX Gene in Various Tissues/Organs

Eight tissues and organs, namely, mature leaves, flower buds, petals, stamens, pistils, leaf buds, flower buds and young stems, were removed from the *L. tulipifera* adult for RNA extraction and expression pattern determination. Quantitative PCR primers were designed according to the full-length cDNA sequence of the *LtuPTOX* gene (Table A1), and the reactions were performed using SYBR Premix Ex Taq enzyme (RR420A, TAKARA, Dalian, China) according to the instructions for the ABI Step-One Plus platform. The relative expression of genes was calculated and plotted with Office 2010 (Microsoft, Washington D.C., USA) and GraphPad 7.0 software (GraphPad Software, Inc. 7825 Fay Avenue, Suite 230 La Jolla, CA 92037, USA).

### 2.6. Expression Levels of the LtuPTOX Gene within the Orange Petal Band at Different Stages

Petals at various developmental stages, from enlargement of flower buds to petals begins to deepen, were used to determine the expression changes of the *LtuPTOX* gene. Material of five petal developmental stages including, S1, female flower buds begin to enlarge, and the flower bud is as hard as a marble; S2, intermediate developmental stage of female flowers, when the flower buds begin to soften; S3, later enlargement phase of the flower bud, when the orange band of the petals appears yellow; S4, the flower buds are preparing to open, and the color of the orange band of the petals begins to deepen; and S5, the peak flowering period of *Liriodendron* flowers, were removed from organism. Orange band tissue at five stages was used for RNA extraction and expression determination according to the manufacturer’s instructions (RR036A and RR420A, TAKARA Dalian, China). The experiments were performed with the ABI Step-One Plus platform (Thermo Fisher Scientific, SC, USA), and then we calculated expression with Microsoft Office software.

### 2.7. Quantification of Chlorophyll and Carotenoid Contents in the Orange Petal Band

We further removed the orange band region of Liriodendron petals at different stages for chlorophyll and carotenoid determination. In vitro tissues were pulverized in liquid nitrogen, and all the materials were standardized to 0.1 g. Then, 10 mL of extraction liquor (80% acetone and 20% absolute ethanol) was added to the centrifuge tube. After 2 days of extraction at 37 °C under dark conditions, when the materials were thoroughly faded, the mixed liquor was centrifuged at 12,000 rpm. Afterwards, the supernatants were collected to determine pigment content. Moreover, we detected the absorbance value at multiple wavelengths (470 nm, 647 nm and 663 nm). Finally, the chlorophyll and carotenoid contents were calculated according to the method of Arnon and Myeong J. K. [34,35]. The equations are provided as follows:

ChlA (mg/g FW) = [(12.7 × (A) − 2.69 × (B))/(D × 1000 × W)] × V,(1)

ChlB (mg/g FW) = [(22.9 × (B) − 4.68 × (A))/(D × 1000 × W)] × V,(2)

Chl (mg/g FW) = [(20.29 × (B) + 8.02 × (A))/(D × 1000 × W)] × V,(3)

CxC (mg/g FW) = [((1000 × (C) − 1.82 × (ChlA) − 85.02 (ChlB))/198)/(D × 1000 × W)] × V.(4)

## 3. Results

### 3.1. Full-Length cDNA Isolation of the LtuPTOX Gene

Six EST fragments annotated as PTOX or IMMUTANS (Table 1) were obtained from the *L. tulipifera* EST database [27] (AAGP, http://jlmwiki.plantbio.uga.edu/aagp). To generate the target sequence of the *LtuPTOX* gene and DEGs associated with petal development, alignments of the 6 fragments were performed with the DEG database at different developmental stages. By perfect alignments and the removal of redundant sequences, a unigene named Liriodend_newGene_31435 was ultimately obtained. We searched for the unigene in the genome-wide sequences of *L. chinense* and found that the Lchi07087 gene was extremely consistent with Liriodend_newGene_31435. Afterwards, a gene-specific primer was designed to isolate the full-length cDNA of *LtuPTOX*.

**Figure A1:**
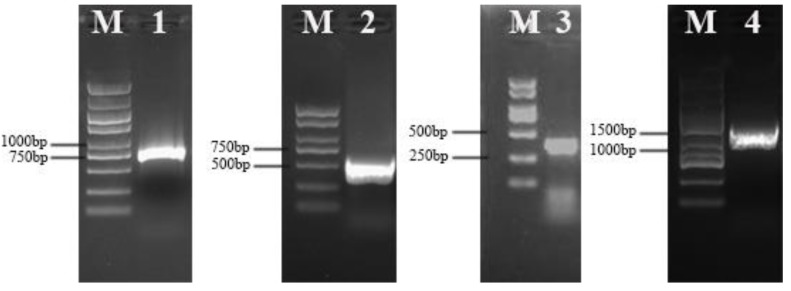
Isolation and detection of the LtuPTOX gene. DNA fragments were detected by 1.5% agarose gel. Lanes 1 ~ 3 represent the intermediate and untranslated regions of 5′ and 3′, respectively. Lane 4 is the open reading frame.

Intermediate and 5′ and 3′ untranslated regions were amplified in vitro under different conditions, and their lengths were 764 bp, 396 bp, and 468 bp (Figure A1). We further assembled the three fragments in silico using DNAstar software, and primers were assigned at both ends of the sequence for full-length isolation. Finally, we obtained the complete sequence of the *LtuPTOX* gene.

### 3.2. In Silico Analysis of the Function and Structure of LtuPTOX Gene

**Figure 2:**
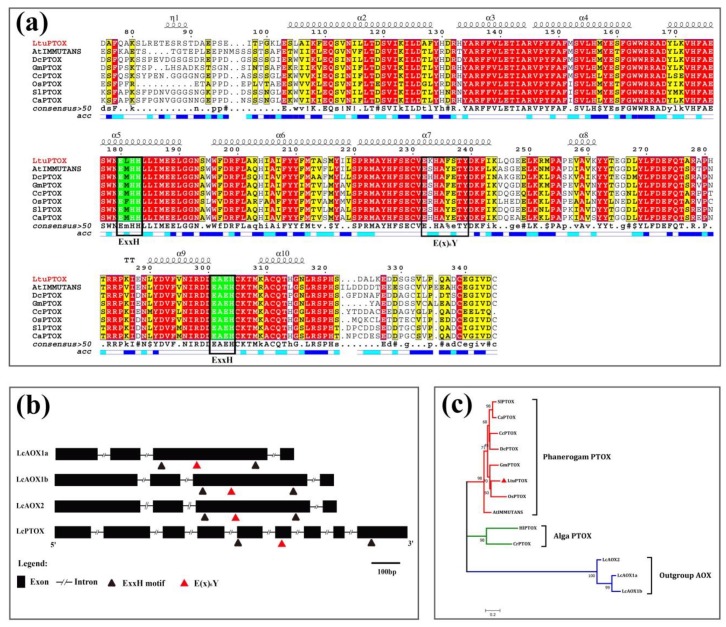
Sequence alignment and phylogenetic analysis of LtuPTOX (**a**), Multiple sequence alignment of PTOXs, where the helixes on top of the lines are α-helixes, blue and white bars indicate the site accessibility of the protein (blue bars represent the amino acids that are accessible, cyan bars represent those with intermediate accessibility, and white bars indicate those that are not accessible. ExxH modules with a green background are the putative di-iron coordination motifs, and the E(x)_6_Y module with black border is considered to be another active site [37]. (GenBank accessions: SlPTOX, NP 001234511.1; CaPTOX, AAG02288.1; CcPTOX, ABB70513.1; DcPTOX, AJE24555.1; GmPTOX, NP_001242139.2; LtuPTOX, MN368606; AtIMMUTANS, AJ004881; OsPTOX, AF085174). (**b**), Gene structure comparison of *Liriodendron* PTOX and AOXs, where rectangles represent exons, lines represent introns, and triangles represent di-iron coordination motifs. (**c**), Phylogenetic analysis based on PTOXs and the *Liriodendron* AOXs (LcAOX1a, MN187966; LcAOX1b, MN187968; and LcAOX2, MN187967; HlPTOX, ABF85789.1; CrPTOX, XP_001703466.1).

The full-length ORF of the LcPTOX gene is 1077 bps long and encodes a 358-amino acid (aa) protein (GenBank ID: MN368606). The relative molecular mass of LtuPTOX is approximately 41.3 kDa, and the theoretical isoelectric point is 5.98, which provides evidence for the faint acidity of the LtuPTOX protein. Multiple sequence alignment of PTOX proteins with several other plants (model and non-model plants across the monocotyledons and dicotyledons) was performed using DNAMAN and ESPrint software; the identity of these PTOXs was approximately 72.57%. The PTOX protein shared a conserved domain from 85 aa to 313 aa (PF01786) with AOX, and “ExxH” Di-iron coordination motifs, which were considered iron binding sites in many other studies [36], were found in this region (Figure 2a). In addition, the “E(x)6Y” motif, another active site [37], was also shared between these two subfamilies as demonstrated in Figure 2a,b. Subsequently, we compared the gene structure of *PTOX* with that of *AOX*, a paralog located in mitochondria based on *Liriodendron* genome sequences, as these two subfamilies were frequently mistakenly placed in the same category in previous studies. The *Liriodendron* AOX protein possesses four exons and three introns, and the PTOX gene possesses nine exons and eight introns. Moreover, the AOX protein contains two “ExxH” motifs, both of which are located in the third exon. However, the two “ExxH” motifs are distributed in the fifth and ninth exons, respectively (Figure 2b).

PTOXs of several other species, including six spermatophytes and two algae, were used for phylogenetic analysis. In order to correct the NJ tree, the LcAOXs were also included as outgroups in this process. Three categories, namely, spermatophyte, alga and outgroup, are clearly displayed in Figure 2c. Moreover, the genetic relationship between OsPTOX and LtuPTOX was closer than that between other pairs, according to the phylogenetic tree.

### 3.3. Transient Transformation and Subcellular Localization of LtuPTOX

**Figure 3:**
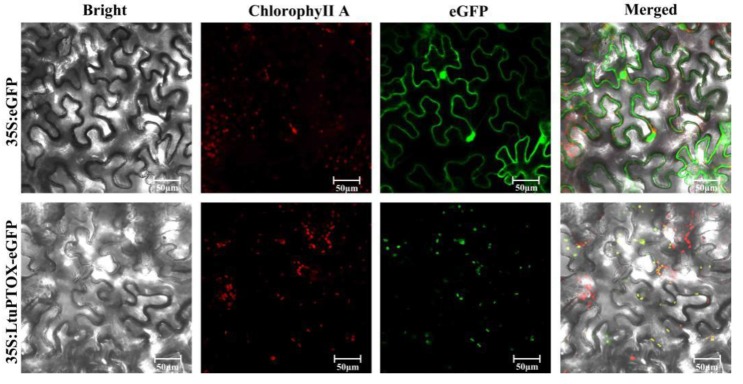
Subcellular localization of *LtuPTOX* in tobacco leaves.

Determining the subcellular localization of a protein is critical in establishing its function. By expressing a fusion gene of *LtuPTOX* and *eGFP* in epidermal cells using tobacco transient expression approaches, we successfully determined the subcellular localization of the LtuPTOX proteins. On the laser confocal microscope platform, green fluorescence of the tobacco cells in the control group, which carried *35S:eGFP* genes, displayed a global expression pattern. However, green signals in the tobacco cells modified by the *35S:LtuPTOX-eGFP* gene showed the same location as chloroplasts in the lower epidermis (Figure 3). Based on these results, the LtuPTOX protein might play a significant role in chloroplasts, which is consistent with the finding of its potential function as a protective factor for chlorophyll in many other studies [21,38,39,40]. However, in other cases, *PTOX* genes also play important roles in nonchlorophyllous tissues [41,42].

### 3.4. Expression Pattern of the LtuPTOX Gene in Various Tissues/Organs

**Figure 4:**
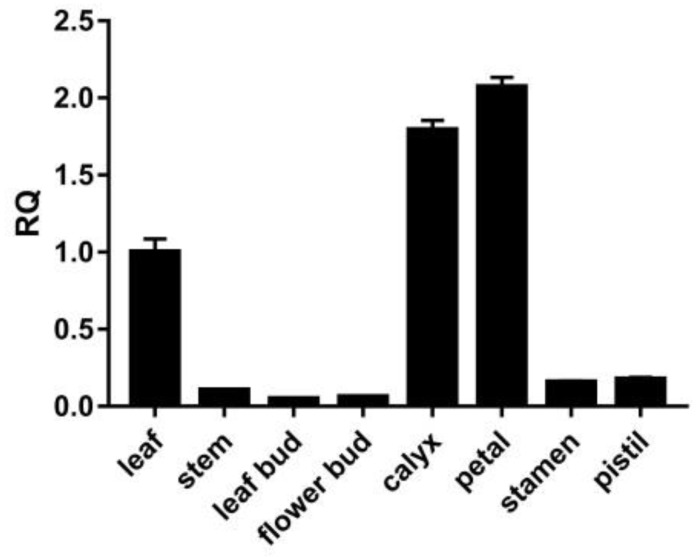
Expression patterns of *LtuPTOX* in eight tissues/organs.

In order to understand the expression pattern of the *LtuPTOX* gene in various tissues and organs, we used qRT-PCR to determine the relative expression of *LtuPTOX*. The qPCR results showed that petals possessed the highest abundance of transcripts, followed by calyxes and leaves (Figure 4). The expression in these three tissues was far greater than that in the other tissues and organs in this assay, which suggested that tissues containing many pigments (e.g., chlorophyll and carotenoids) might also contain a large number of *LtuPTOX* transcripts. In many cases, PTOX acts as a safety valve in the photosynthetic system [43]; however, we detected very high enrichment in tissues in which green pigments gradually faded and even in nonchlorophyllous tissues, such as the petals. Hence, we inferred that *LtuPTOX* is more than just a safety valve for chlorophyll.

### 3.5. LtuPTOX Involved in Carotenoid Metabolism and Orange-Band Formation on the Tepal

**Figure 5:**
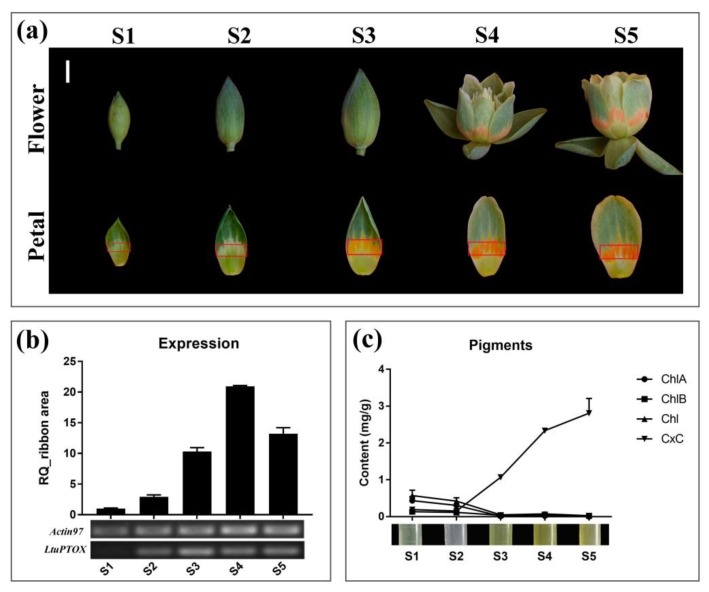
Changes in pigment content and gene expression in the orange bands of Liriodendron petals during different developmental stages. (**a**), the different developmental stages, i.e., S1, female flower buds begin to enlarge, and the flower bud is as hard as a marble; S2, intermediate developmental stage of female flowers, when the flower buds begin to soften; S3, later enlargement phase of the flower bud, when the orange band of the petals appears yellow; S4, the flower buds are preparing to open, and the color of the orange band of the petals begins to deepen; and S5, the peak flowering period of *Liriodendron* flowers, when pollination and fertilization begin. The orange band marked by a red box on each of the petals was used for RNA extraction and pigment determination, and the bar is 1 cm; (**b**), Changes in the expression level of the LtuPTOX gene from S1 to S5. (**c**), Changes of the four pigments content in different developmental stages, including Chlorophyll A (ChlA), Chlorophyll B (ChlB), total Chlorophyll (Chl), and Carotenoids (CxC).

To thoroughly investigate the relationship between *LtuPTOX* expression and flower color changes in *L. tulipifera*, orange petal band tissues at different developmental stages were used for RNA extraction and expression detection (Figure 5a). Although the orange band region on the petals did not initially appear to be green, the results of both quantitative and semiquantitative PCR demonstrated upward trends as the color of the petals deepened (Figure 5b). In a previous study, discoloration of tomato fruits was shown to be caused by a loss-of-function mutation in the PTOX allele, and then the transformation of phytoene and ζ-carotene was blocked [19,44]. We further quantified the chlorophyll and carotenoid contents in orange petal bands at different developmental stages. The chlorophyll content decreased gradually with petal development and reached zero in the later flower bud enlargement phase (S3). In striking contrast to chlorophyll, carotenoids accumulated rapidly beginning in the S3 period (Figure 5c). The change in *LtuPTOX* expression was highly consistent with the carotenoid content accumulation, and the same pattern was also found in *Arabidopsis* seedlings [19] and tomato fruits [44]. In summary, LtuPTOX is a critical gene involved in the formation of orange bands on petals.

## 4. Discussion

In this study, a putative *LtuPTOX* encoding a 358-aa protein was derived from *L. tulipifera* through multiple alignments and gene screening against the RNA-seq DEG database as well as the complete genome sequence of *L. chinense*. An AOX conserved domain from 85 aa to 313 aa was identified in the LtuPTOX protein sequence, which indicated its similarity to the PTOX and AOX gene sequences. *PTOX* genes are often compared with *AOX* genes, as they have similar sequences and share the same conserved domains. The results of evolutionary analysis showed that these genes were derived from the same ancestral gene [45,46]. Moreover, an active site, “E(x)6Y”, detected by Nakamura was also found in the conserved domain in both subfamilies [37], as were two di-iron coordination motifs named “ExxH”, although they were quite different in gene structure and location [9]. This structural consistency might lead to a strong similarity in function in many cases, such as the functional redundancy of the two subfamily proteins of *A. thaliana* detected in chloroplasts [47]. In contrast to the respiratory ETC in mitochondria, the chlororespiration ETC carries a distant terminal oxidase, which is able to transfer electrons from NADH/NADPH to plastoquinone (PQ) [48].

Based on previous studies, PTOX is a terminal oxidase involved in chlororespiration that regulates the redox state of the PQ pool [49] by transferring excess electrons to O2 in order to maintain the relative redox balance in the photosynthetic ETC. In this study, chloroplast subcellular localization of LtuPTOX was observed in tobacco leaves, which was consistent with the safety valve function of this gene in photorespiration and stress responses [14,39,50]. The same gene participates in the synthesis of carotenoids in *A. thaliana* and *Solanum lycopersicum* [19,41], even though tomato fruits do not contain chloroplasts. In addition, the dual role of PTOX was further characterized in different plastids of these two model plants [9,13]. Besides, the high expression level of LtuPTOX in colored organs and tissues provided an additional line of evidence.

Plastids are of great difference to plant cells depending on the pigments or other metabolites contained in higher plants. In addition to chloroplasts, other well-known plastids, i.e., chromoplasts, etioplasts, leukoplasts, amyloplasts, proteinoplasts, etc., are found in different vegetables. However, these various types of plastids are able to rapidly interconvert under special conditions [51]. PTOX is identified as plastid terminal oxidase which would transfer electrons from PQ to O_2_ in the synthesis of chlorophyll in chloroplasts as well as carotenoids in chromoplasts. In this manner, we inferred that PTOX acting as an electron mediator might be shared among various plastids in plants.

Finally, we illustrated the fact that the expression of *LtuPTOX* increased in the orange-band region during the period of tepal development. We also demonstrated the complete synchronicity of gene expression and carotenoid accumulation during this period, especially the abrupt increase that occurred from S2 to S3. Consistent with our results, novel pathways of electron transport mediated by AtPTOX were recently found in etioplasts of *Arabidopsis* [52]. Kambakam found that AtPTOX coupling with PGR5 and the NDH complex caused electron transfer from PDS and ZDS to PQH2 in the carotenoid biosynthesis pathway under dark conditions. Despite the absence of virus-induced gene silencing (VIGS) methods in *Liriodendron* that could be used to further explore this process, we showed at both the transcriptional and metabolic levels that LtuPTOX is involved in carotenoid metabolism and orange band formation on the tepals of *L. tulipifera*, which will enable an understanding of the color formation mechanism of *L. tulipifera*.

## 5. Conclusions

In this study, we identified a putative LtuPTOX gene derived from *L. tulipifera* by multiple database selection. Combined with a series of transcriptional and metabolic detection, we inferred the *LtuPTOX* gene might participate in carotenoid synthesis and orange-band of the petals. In conclusion, this study will lay a foundation for further uncovering the mechanism of flower color of *L. tulipifera*.

## Figures and Tables

**Table 1:** Screening and identification of LtuPTOX from multiple *Liriodendron* database.

AAGP Gene NO. ^1^	RNA-seq Dataset	Gene ID ^2^	Annotation
gnl|Liriodendron|b4_c3586	Liriodend_newGene_31435	Lchi07087	PTOX/IMMUTANS
gnl|Liriodendron|b4_c15469			
gnl|Liriodendron|b4_c23296			
gnl|Liriodendron|b4_c24190			
gnl|Liriodendron|b4_s124823			
gnl|Liriodendron|b4_rep_c78233			

^1^ AAGP data base: http://jlmwiki.plantbio.uga.edu/aagp; ^2^ Genome Database: ftp://ftp.cngb.org/pub/CNSA/CNP0000295/CNS0044063/CNA0002404/.

## Appendix A

**Table A1:** Primer sequences used in the article.

Primer Name	Primer Sequence	TM/°C
nG31435-F	AACCCAGGAAGACCATCTCC	57
nG31435-R	GGAGCTCTTGCTGTTTGAA	54
5GSP1	CAAATAATCAGCTCTTCTCCAC	55.6
5GSP2	CCTCCACGATAACTTCTTCCTCC	61
3GSP1	GTTTGACCGTTTTCTTGCTC	54.6
3GSP2	GGAGAAGAATTGAAAAGAATGCCTG	58.1
LtuPTOX-F	TACTCAGCAACGAATAAACC	52.2
LtuPTOX-R	TTGGATAAATATGGACCCCT	52.9
p1302-LtuPTOX-TF	actcttgaccatggtagatctATGACTACAAGATCAACATCTCTCTCTTC	60.6
p1302-LtuPTOX-TR	aagttcttctcctttactagtTTCTTGTTTCCTTTCATGAGGGG	62.4
35S primer	GACGCACAATCCCACTATCC	56
LtuPTOX-qF	CTGTTCTGCACATGTACGAGA	57.8
LtuPTOX-qR	GCAAGAAAACGGTCAAACCAC	57.9
Actin97-qF	TTCCCGTTCAGCAGTGGTCGTGGTCG	58
Actin97-qR	TGGTCGCACAACTGGTATCG	58
